# Genome-Wide Selection Signatures and Human-Mediated Introgression Events in *Bos taurus indicus*-influenced Composite Beef Cattle

**DOI:** 10.3389/fgene.2022.844653

**Published:** 2022-05-30

**Authors:** Seyed Milad Vahedi, Siavash Salek Ardestani, Kian Pahlevan Afshari, Seyed Mohammad Ghoreishifar, Sima Moghaddaszadeh-Ahrabi, Mohammad Hossein Banabazi, Luiz Fernando Brito‬‬

**Affiliations:** ^1^ Department of Animal Science and Aquaculture, Dalhousie University, Truro, NS, Canada; ^2^ Department of Animal Science, Science and Research Branch, Islamic Azad University, Tehran, Iran; ^3^ Department of Animal Sciences, Islamic Azad University, Varamin-Pishva Branch, Varamin, Iran; ^4^ Department of Animal Science, University College of Agriculture and Natural Resources, University of Tehran, Karaj, Iran; ^5^ Department of Animal Science, Faculty of Agriculture and Natural Resources, Islamic Azad University, Tabriz Branch, Tabriz, Iran; ^6^ Department of Animal Breeding and Genetics (HGEN), Centre for Veterinary Medicine and Animal Science (VHC), Swedish University of Agricultural Sciences (SLU), Uppsala, Sweden; ^7^ Department of Animal Sciences, Purdue University, West Lafayette, IN, United States

**Keywords:** indicine, taurine, composite beef cattle, population genetics, introgression, recent selection signatures

## Abstract

Genetic introgression from interbreeding hybridization of European *Bos taurus taurus* (EBT) and Indian *Bos taurus indicus* (IBI) cattle breeds have been widely used to combine the climatic resilience of the IBI cattle and the higher productivity of EBT when forming new composite beef cattle (CB) populations. The subsequent breeding strategies have shifted their initial genomic compositions. To uncover population structure, signatures of selection, and potential introgression events in CB populations, high-density genotypes [containing 492,954 single nucleotide polymorphisms (SNPs) after the quality control] of 486 individuals from 15 cattle breeds, including EBT, IBI, and CB populations, along with two *Bos grunniens* genotypes as outgroup were used in this study. Then, in-depth population genetics analyses were performed for three CB breeds of Beefmaster, Brangus, and Santa Gertrudis. Neighbor-joining, principal components, and admixture analyses confirmed the historical introgression of EBT and IBI haplotypes into CB breeds. The f_dM_ statistics revealed that only 12.9% of CB populations’ genetic components are of IBI origin. The results of signatures of selection analysis indicated different patterns of selection signals in the three CB breeds with primary pressure on pathways involved in protein processing and stress response in Beefmaster, cell proliferation regulation and immune response in Brangus, and amino acids and glucose metabolisms in Santa Gertrudis. An average of >90% of genomic regions underlying selection signatures were of EBT origin in the studied CB populations. Investigating the CB breeds’ genome allows the estimation of EBT and IBI ancestral proportions and the locations within the genome where either taurine or indicine origin alleles are under selective pressure. Such findings highlight various opportunities to control the selection process more efficiently and explore complementarity at the genomic level in CB populations.

## Introduction

Various cattle breeds with particular phenotypes and genetic backgrounds have been artificially selected worldwide mainly for agricultural purposes, such as milk yield, meat production, and climatic resilience (Bélanger and Pilling, 2019; Freitas et al., 2021). Two main types of modern cattle breeds of *Bos taurus taurus* (humpless taurine) and *Bos taurus indicus* (humped indicine) are the result of two domestication processes in the Fertile Crescent and the Indus Valley, respectively (Hiendleder et al., 2008; Bickhart et al., 2016). Population genetic studies based on genomic single-nucleotide polymorphism (SNP) data identified three major groups of cattle, comprising of Asian indicine, Eurasian taurine, and African taurine (Bovine HapMap Consortium, 2009; McTavish et al., 2013). However, a prominent whole-genome sequencing analysis of domestic cattle populations demonstrated that the worldwide cattle population could be classified into five continental groups based on Y-chromosome haplotypes and autosomal variants: European taurine, Eurasian taurine, East Asian taurine, Chinese indicine, and Indian indicine (Chen et al., 2018). In contrast, composite cattle breeds have been developed by human-mediated crossing of two or more breeds, in specific proportions, to combine their desirable and complementary traits into one breed (Gregory and Cundiff, 1980; Gregory et al., 1994). Once the composite breed is formed, intensive selection programs, mainly for traits valued by breeders, are applied to improve the rates of genetic gain and animal productive efficiency (Heaton et al., 2002; Grigoletto et al., 2020).

Over the last half-century, some *Bos taurus indicus*-influenced composite beef cattle (CB) breeds, such as Beefmaster, Brangus, and Santa Gertrudis, have been developed in the US to combine the climatic resilience of the Indian *Bos taurus indicus* cattle (IBI) and the higher productivity of European *Bos taurus taurus* (EBT) (Buzanskas et al., 2017; Gregory and Cundiff, 1980). Beefmaster is the first American composite breed which was developed through crossing between Brahman, Shorthorn, and Hereford on the Lasater Ranch in Falfurrias, Texas in 1908. The pedigree-estimated breed composition in Beefmaster is expected to be ½ Brahman, ¼ Hereford, and ¼ Shorthorn (Warwick, 1958). Brangus cattle was derived from crosses between Angus and Brahman cattle in Oklahoma, Mississippi, Texas, and Louisiana in the 1930s and the breed’s genome content of ⅜ Brahman and ⅝ Angus is expected (International Brangus Breeders Association; https://gobrangus.com/). Santa Gertrudis cattle was developed on the King Ranch in Kingsville, Texas, by experimental crossbreeding between Shorthorn and Brahman cattle between 1910 and 1920. This breed is expected to have a genetic composition of ⅜ Brahman and ⅝ Shorthorn (Rhoad, 1949; Warwick, 1958).

One of the primary sources of genetic variability, particularly in composite breeds, is adaptive variation transmitted to the breed by introgression, a phenomenon referred to as “adaptive introgression” (Hedrick, 2013). Therefore, almost all the variants present in the populations of CB breeds will have been of introgressed origin from IBI and EBT breeds. The availability of whole-genome DNA information on a large number of individuals enables assigning ancestry along a chromosome and identify ancestry genomic regions introgressed into hybrid breeds using high-throughput genomic data (Malinsky et al., 2021). Introgression analysis of CB cattle breeds represents an appealing model for understanding the genomic consequences of crossbreeding with the goal of improving traits of interest.

The processes of domestication, breed formation, and subsequent selection have left detectable footprints within the genome of CB cattle breeds (Paim et al., 2020; Singh et al., 2020). Some of these footprints of selection (also known as selection signatures) reflect the historical selection during cattle domestication, whereas some represent selection within the past few generations for economically important traits, including meat production or environmental adaptation (Decker et al., 2014). Regarding the recent crossbreeding events in CB breeds, attention must be given to the more recent systematic, organized selection following the breed formation than old selective sweeps. Meanwhile, several extended haplotype homozygosity (EHH)-related statistics have been developed to detect more recent selection events (Sabeti et al., 2002). Moreover, the results of multiple EHH-based tests can be integrated into different composite measures of selection (Lotterhos et al., 2017). One of the well-known composite measures is the de-correlated composite of multiple signals (DCMS), proposing higher resolution and power of detecting selection signals compared to most single statistics (Ma et al., 2015) and composite measures of selection footprints (Lotterhos et al., 2017).

This study uses genomic data generated from the Illumina BovineHD SNP Beadchip (San Diego, CA, United States) to trace the ancestry components and understand the introgression processes that formed the currently observed diversity of CB breeds. Moreover, we applied the DCMS method to explore recent selection signatures in the autosomal genome of CB breeds to investigate the possible determinants of adaptation and production.

## Materials and Methods

### Data Collection and Quality Control

Genotype data of 15 cattle breeds (*N* = 486), including nine EBT, three IBI, and three CB breeds, along with two *Bos grunniens* samples as outgroup was obtained from the WIDDE database (Sempéré et al., 2015). Only breeds genotyped with the Illumina Bovine HD Genotyping BeadChip (www.illumina.com; San Diego, CA, United States) and that had at least 10 individuals were included in this study. Table 1 presents the cattle breeds, their abbreviations, and number of individuals included in this study. Quality control of 732,993 SNP was performed using the *VCFtools* 0.1.16 (Danecek et al., 2011) for all genotypes. Markers with minor allelic frequency <0.01, SNP calling rate <0.90, extreme departure from Hardy-Weinberg equilibrium *p*-value < 10^–7^, and SNPs located on non-autosomal chromosomes were removed. Furthermore, samples with a genotype call rate of less than 90% were discarded from downstream analyses. After the quality control, 492,954 SNPs from 488 animals remained for further analyses.

**TABLE 1:** Descriptive statistics for the studied cattle breeds.

Type	Breed	Abbreviation	N	Total
European *Bos taurus* (EBT)	Angus	ANG	42	315
	Brown Swiss	BSW	22	
	Charolais	CHL	37	
	Guernsey	GNS	21	
	Herford	HFD	28	
	Holstein	HOL	60	
	Jersey	JER	34	
	Limousin	LMS	50	
	Piedmontese	PMT	21	
Indian *Bos taurus indicus* (IBI)	Brahman	BRM	46	104
	Gyr	GYR	27	
	Nellore	NEL	31	
Composite beef cattle (CB)	Beefmaster	BMA	23	67
	Brangus	BRG	12	
	Santa Gertrudis	SGT	32	
*Bos grunniens*	Yak	OUT	2	2

### Population Structure and Phylogenetic Tree

Principal component analysis (PCA) was used to estimate genetic relationships and population structure. PCA was conducted using the PLINK 2.0 software (Purcell et al., 2007). Subsequently, a 3D plot of principal components was constructed using a custom-made Python 3.8 (http://www.python.org) script (https://github.com/Siavash-cloud/3D-PCA-plot). The phylogenetic analysis was performed using the neighbor-joining approach in VCF-kit 0.2.9 (Cook & Andersen, 2017). Visualization of the phylogenetic analysis was based on rooting of the outgroup in *FigTree* 1.4.3 (http://tree.bio.ed.ac.uk/software/figtree). Maximum likelihood analysis of population structure of the studied cattle breeds was conducted using *Admixture* 1.3 (Alexander et al., 2009) for K values ranging from 2 to 20 with 10 iterations per K value. The *Admixture* software (Alexander et al., 2009) uses a cross-validation procedure to estimate the most likely number of ancestral populations (K). The cross-validation error estimates were plotted using the *ggplot2* package (Villanueva and Chen, 2019) in the R 4.0.5 software (R Core Team, 2020) to compare the K values.

### Genomic Introgression Analyses

Patterson’s D (Green et al., 2010) and the related estimate of admixture fraction f, referred to as f_4_-ratio (Patterson et al., 2012) were estimated using the *Dtrios* program implemented in the *Dsuite* package (Malinsky et al., 2021) to assess the evidence for gene flow from EBT and IBI breeds into CB populations. These two statistics are applied to assess correlations of allele frequencies across populations (Patterson et al., 2012). These methods can be successfully used for learning about hybridization and introgression events within groups of closely related populations (Patterson et al., 2012; Pease and Hahn, 2015). In the Patterson’s D and f_4_-ratio statistics, a simple explicit phylogenetic tree model is fitted to a quartet of populations, and a formal test for a history of admixture is performed in that context (Patterson et al., 2012). The D and f_4_-ratio statistics were applied to biallelic SNPs across four populations: P1, P2, P3, and OUT, related by the rooted tree [(P1,P2), P3, OUT], where yak (*Bos grunniens*) was used as the outgroup (OUT) to test if the P1 (EBT cattle) and P2 (IBI cattle) shared more alleles at the SNP level with a candidate introgressor—P3, including the three CB breeds. The site patterns were ordered as follows: BBAA represents P1 and P2 sharing the derived allele, ABBA referred to P2 and P3 sharing the derived allele, and BABA to P1 and P3 sharing the derived allele. Under the null hypothesis, which assumes no gene flow, the ABBA and BABA patterns are expected to occur due to incomplete lineage sorting with equal frequencies, and a significant deviation from that expectation is consistent with introgression between P3 and either P1 or P2. For more details, see Patterson et al. (2012) and Durand et al. (2011). Therefore, for all n biallelic sites, nABBA , nBABA , and nBBAA were calculated as follows (Patterson et al., 2012):

$$nABBA=\overset{n}{\underset{i=1}{\sum }}\left(1-{\hat{p}}_{i1}\right){\hat{p}}_{i2}{\hat{p}}_{i3}\left(1-{\hat{p}}_{iO}\right)+{\hat{p}}_{i1}\left(1-{\hat{p}}_{i2}\right)\left(1-{\hat{p}}_{i3}\right){\hat{p}}_{iO}$$

$$nBABA=\overset{n}{\underset{i=1}{\sum }}{\hat{p}}_{i1}\left(1-{\hat{p}}_{i2}\right){\hat{p}}_{i3}\left(1-{\hat{p}}_{iO}\right)+\left(1-{\hat{p}}_{i1}\right){\hat{p}}_{i2}\left(1-{\hat{p}}_{i3}\right){\hat{p}}_{iO}$$

$$nBBAA=\overset{n}{\underset{i=1}{\sum }}{\hat{p}}_{i1}{\hat{p}}_{i2}\left(1-{\hat{p}}_{i3}\right)\left(1-{\hat{p}}_{iO}\right)+\left(1-{\hat{p}}_{i1}\right)\left(1-{\hat{p}}_{i2}\right){\hat{p}}_{i3}{\hat{p}}_{iO}$$

where ${\hat{p}}_{i1}$ , ${\hat{p}}_{i2}$ , ${\hat{p}}_{i3}$ , and ${\hat{p}}_{iO}$ are the derived allele frequency estimate at site i in P1, P2, P3, and OUT, respectively. Based on Patterson et al. (2012)’s definition, the Dsuit package applies OUT as the fourth population, not necessarily an outgroup; for more details, see Malinsky et al. (2021). Therefore, the implicit assumption that the outgroup population is fixed for the ancestral allele and is necessarily used in that case is no longer applicable (Malinsky et al., 2021). Patterson’s D statistics was calculated by $D=\frac{\sum_{i=1}^{n}\left({\hat{p}}_{i2}-{\hat{p}}_{i1}\right)\left({\hat{p}}_{i3}-{\hat{p}}_{iO}\right)}{\sum_{i=0}^{n}\left({\hat{p}}_{i2}+{\hat{p}}_{i1}-2{\hat{p}}_{i2}{\hat{p}}_{i1}\right)\left({\hat{p}}_{i3}+{\hat{p}}_{iO}-2{\hat{p}}_{i3}{\hat{p}}_{iO}\right)}$ (Patterson et al., 2012). To calculate the f_4_-ratio, P3 was split into two subsets, P3a and P3b, and Dsuite randomly sampled from P3 alleles at each SNP. Then, f_4_-ratio was calculated using f_4_-ratio $=\frac{\sum_{i=1}^{n}\left({\hat{p}}_{i3a}-{\hat{p}}_{iO}\right)\left({\hat{p}}_{i2}-{\hat{p}}_{i1}\right)}{\sum_{i=1}^{n}\left({\hat{p}}_{i3a}-{\hat{p}}_{iO}\right)\left({\hat{p}}_{i3b}-{\hat{p}}_{i1}\right)}$ (Patterson et al., 2012).

To localize the introgressed loci from the breeds with the highest Z-transformed D (Z(D)) and f_4_-ratio values, the *Dinvestigate* software of the *Dsuite* package (Malinsky et al., 2021) and f_dM_ statistics were applied. f_dM_ is a conservative version of the f statistic that is particularly appropriate for analysis of small genomic windows (Malinsky et al., 2018). The f_dM_ statistics (Malinsky et al., 2015) was estimated using a sliding window size of 10 SNPs and a step size of two SNPs (Wang et al., 2020) as follows:

$$f_{dM}=\frac{S\left(P1,P2,P3,OUT\right)\;}{-S\left(Pd,P2,Pd,OUT\right)}$$

Where S(P1,P2,P3,OUT) stands for the numerator of Patterson’s D, and −S(Pd,P2,Pd,OUT) is equivalent to the f_dM_ numerator when Pd=P1 and Pd=P3 , depending on which of $P_{1}$ or $P_{2}$ populations has the higher frequency of the derived allele. Under the null hypothesis, i.e., no introgression, the f_dM_ value is symmetrically distributed around zero, and it can equally quantify shared variation between P3 and P2 (positive values) or between P3 and P1 (negative values) (Malinsky et al., 2021).

### Analysis of Recent Signatures of Selection

To detect recent signatures of selection in each CB breed, three EHH-related within-population signatures of selection tests, including integrated haplotype score (iHS; Voight et al., 2006), integrated haplotype homozygosity pooled (iHH12; Torres et al., 2018), and nSL (Ferrer-Admetlla et al., 2014) were applied using *selscan* 2.0.0 software (Szpiech and Hernandez, 2014). Prior to analyses, missing genotypes were removed using *VCFtools* 0.1.16 (genotype call rate = 100%) since selscan cannot handle missing genotypes. Genotype imputation was not performed because there were not enough genotyped individuals to accurately perform imputation, especially when considering that the CB populations have high genetic diversity. The nSL procedure calculates the SL statistic that measures the length of a segment of haplotype homozygosity in terms of segregating sites (Ferrer-Admetlla et al., 2014). Then, the three statistics of iHS, iHH12, and nSL were combined into a single DCMS framework. The approach of combining multiple selection signals in the DCMS framework has been previously performed in several studies (e.g., Yurchenko et al., 2018; Ghoreishifar et al., 2020). DCMS combines *p*-values produced by multiple statistics for each locus into a single measure considering the correlation between the statistics (Lotterhos et al., 2017). The DCMS statistic is calculated at the position *l* as follows (Ma et al., 2015):

$$DCMS_{l}=\overset{n}{\underset{t=1}{\sum }}\frac{\log\left[\frac{1-p_{lt}}{p_{lt}}\right]}{\sum_{i=1}^{n}\left|r_{it}\right|}$$

where $p_{lt}\;$ shows the *p*-value at position l for statistic t ; $r_{it}\;$ refers to the correlation between the test statistic of the i th and t th methods, and n is the total number of test statistics combined in the DCMS. The expression $\frac{1}{\sum_{i=1}^{n}\left|r_{it}\right|}$ is called weight factor, which ranges from $\frac{1}{n}\;\;$ to 1.

Therefore, within a given CB breed, for each statistic of iHS, iHH12, and nSL, genome-wide *p*-values were calculated based on fractional ranks using the *stat_to_pvalue* function of the R *MINOTAUR* package (Verity et al., 2017). Fractional rank *p*-values were estimated using a right-tailed test for all statistics (two.tailed = FALSE, right. tailed = TRUE); then, DCMS statistics were calculated using the *DCMS* function of the R *MINOTAUR* package (Verity et al., 2017). DCMS values were then fitted to a normal distribution using the robust linear model (*rlm* function) of the *MASS* R package (Venables & Ripley, 2013) and model = rlm (dcms ∼ 1), where the dcms object is a vector containing the raw DCMS values. The outputs of the fitted model, including mean and standard deviation, were used by the *pnorm* R function to calculate the *p*-values of the DCMS statistics (lower.tail = FALSE, log. p = FALSE). Finally, to control for multiple testing false discovery rate (FDR) among rejected null hypotheses, the DCMS *p*-values were transformed to the corresponding *q*-values using the *qvalue* R function and the Benjamini and Hochberg method (Benjamini and Hochberg, 1995).

We extracted the f_dM_ value of genomic regions harboring SNPs with significant selection signals (*q*-value < 0.05). The average f_dM_ values of regions harboring each significant SNP under selection were used to identify the ancestry. To show the overlaps between signatures of selection and introgression analyses, graphical visualization of bovine chromosomes was applied using the *chromoMap* 0.3.1 package (Anand and Lopez, 2022) in the R software (R Core Team, 2020).

### Gene Annotation

After z-transforming the f_dM_ values (Z(f_dM_)), genomic regions ranked the highest and lowest 95 percentile of Z(f_dM_) values were considered as putative introgressed genomic regions into CB breeds. This threshold was set since significant Patterson’s D values indicated that on average 6.39% of bovine genome was introgressed into the CB breeds. A similar threshold level and approach have been previously applied by Barbato et al. (2020) and Morales-Cruz et al. (2021). To locate the putative regions under selection, chromosome intervals harboring SNPs with a *q*-value < 0.05 were considered as statistically significant intervals, and boundaries of each interval were defined by the locations of the first flanking SNPs exhibiting a *q*-value > 0.10. The advantage of this approach is that fewer candidate genes are obtained for the selection peaks (Yurchenko et al., 2018). Then, protein-coding genes were extracted from the significant regions based on the UMD 3.1 bovine reference genome assembly (Zimin et al., 2009). Manhattan plots of the results were created using the R package *CMplot* 3.6.2 (Yin et al., 2021).

### Protein-Protein Interaction Network Construction and Functional Enrichment Analysis

The genes identified for each CB breeds were separately applied for functional enrichment and protein-protein interaction (PPI) network analyses. Prior to the analyses, duplicated genes were removed from the genes list of each breed. Functional profiling (g:GOSt) of *gProfiler* software (Raudvere et al., 2019) was used to determine terms of Gene Ontology (GO) biological processes (GO:BP) or biological pathways in the Kyoto Encyclopedia of Genes and Genomes (KEGG), WikiPathways, and Reactome databases in which the candidate genes identified in the introgression study were statistically overrepresented. The functional enrichment analysis was conducted based on the reference gene list of *Bos taurus*. Regarding candidate genes found in selection signature analyses, PPI network and functional enrichment analyses were conducted using the *Cytoscape* 3.8.2 software (Shannon et al., 2003) and the reference gene list of *Bos taurus*. The *stringApp* (Doncheva et al., 2018) of *Cytoscape* was used to import and augment networks from the STRING protein database (https://string-db.org/). Physical interactions with the confidence cutoff ratio of 0.4 (default setting) and a maximum of 20 additional interactions were tested. The *stringApp* predicts interactions based on co-expression analysis, evolutionary signals across the cattle genome, automatic text-mining, and orthology-based evidence transfer across organisms. To identify biological pathways enriched in the candidate gene lists, gene enrichment analysis was performed for GO:BP, KEGG, WikiPathways, and Reactome terms. The FDR value < 0.05 was considered as the threshold for identifying the overrepresented terms in all functional enrichment analyses.

## Results

A total of 488 cattle from a publicly-available database were included in this study (Table 1). After quality control, 492,954 SNPs from all animals remained for further analyses. The average ± standard deviation of inter-marker distance of the different breeds was 4.9 ± 8.0 Kb, and the minimum and maximum distance between SNPs were 0.1 and 36.6 Kb, respectively.

### Population Genetic Structure

**FIGURE 1:**
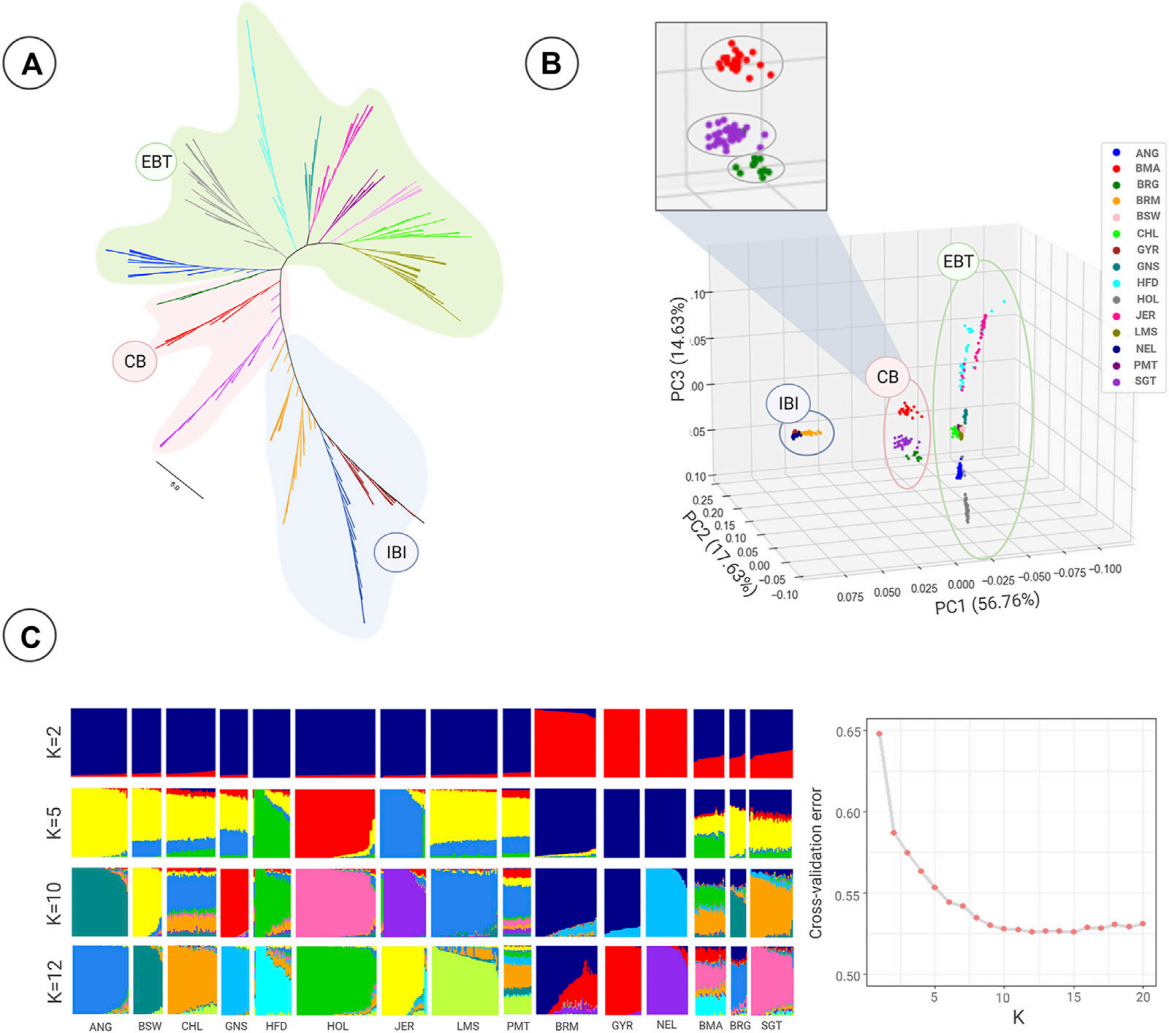
Population structure and relationship of European *Bos taurus taurus* (EBT), Indian *Bos taurus indicus* (IBI), and composite beef cattle (CB) breeds tested in this study using genome-wide SNPs. **(A)** A neighbor-joining phylogenetic tree was constructed using whole-genome SNP data. The scale bar represents pairwise distances between different individuals. Colors reflect the different geographic regions of samples. **(B)** Principal component analysis showing PC1 against PC2 and PC3. **(C)** Model-based clustering of 15 cattle breeds using admixture analysis with the assumed number of ancestries of 2, 5, 10, and 12. The linear plot shows the cross-validation error as a function of K for admixture analysis. Abbreviations of all breeds are given in Table 1.

The phylogenetic analysis illustrated EBT and IBI breeds in two separate main branches (Figure 1A). We noted that CB breeds were located at intermediate positions between these two major clades, as expected. The CB breeds gathered in a paraphyletic pattern, most likely due to the gene flow between IBI and CB. The Brangus was clustered in one clade with the Angus breed, suggesting a closer genetic relationship with EBT cattle, particularly Angus, compared to other populations (Figure 1A). In contrast, Santa Gertrudis demonstrated more intimate genetic relationships with the Brahman breed from the IBI group. The Beefmaster breed was also located between Santa Gertrudis and Brangus clades, with a slight affinity to the EBT clades.

Similar population affinities were obtained based on the PCA results, in which a clear genetic structure with samples from each geographical region clustering together was observed (Figure 1B). The first component (PC1 = 56.76%) was driven by the difference between three large clusters of EBT, IBI, and CB. However, within the CB cluster, a separation was found between Beefmaster, Brangus, and Santa Gertrudis breeds along the second (PC2 = 17.63%) and third (PC3 = 14.63%) principal components.

The ancestral lineage compositions of 15 cattle breeds from global populations are shown in Figure 1C. The K value, representing the number of ancestral populations, indicated that the lowest cross-validation error is K = 12. However, with regards to the history of the different populations and the practice of crossbreeding, we chose to plot the admixture results from two assumed ancestries (K = 2) to optimal K of 12 (Figure 1C). In K = 2, the samples were split into two groups: 1) pure EBT or IBI animals and 2) crossbred CB breeds (combination of the two ancestral populations). Results revealed the presence of EBT and IBI breeds admixture in all three CB breeds populations, confirming the principal component analysis plot. On average, Beefmaster showed 0.71 ± 0.04 and 0.29 ± 0.04 of EBT and IBI ancestry. In contrast, the average EBT and IBI ancestral contribution in Brangus was estimated at 0.69 ± 5 and 0.31 ± 0.05, respectively. In Santa Gertrudis, we estimated an average of 0.64 ± 0.03 and 0.36 ± 0.03 of EBT and IBI ancestry, respectively. Among the IBI breeds, Gyr had the highest percentage of IBI ancestry in the Beefmaster genome (0.21 ± 0.04, K = 12), whereas Brahman was the most introgressed IBI breed to Brangus genome (0.18 ± 0.05, K = 12). Furthermore, Nellore achieved the highest level of admixture in Santa Gertrudis among IBI breeds (0.07 ± 0.04, K = 12). Among EBT breeds, Hereford (0.28 ± 0.06, K = 8) and Angus (0.59 ± 0.05, K = 12) had the highest genomic contribution in Beefmaster and Brangus, respectively. Moreover, Hereford (0.16 ± 0.06, K = 12) gained the most significant introgression among EBT breeds in the Santa Gertrudis genome.

### Detection of Introgression

Among 455 possible breed trios, we found 306 trios with significant Z(D) values (*p* < 0.05). Among them, 38, 44, and 56 trios revealed the significant admixture in Beefmaster, Brangus, and Santa Gertrudis breeds. We also estimated 230 trios with ${\text{f}}_{4}‐\text{ratio }$ > 0.1%, among which 37, 36, and 51 trios demonstrated the gene flow from EBT and IBI populations to Beefmaster, Brangus, and Santa Gertrudis breeds, respectively.

### Beefmaster

Among 29 significant Z(D) values indicating introgression events with EBT breeds, the highest value belonged to the Hereford breed while the Piedmontese was considered as P1 ((((PMT,BMA),HFD),OUT); D = 0.100; Z(D) = 30.020). This result was confirmed by ${\text{f}}_{4}‐\text{ratio}$ as the highest ${\text{f}}_{4}‐\text{ratio}$ value belonged to Hereford ((((((((ANG,HFD),BMA),OUT); ${\text{f}}_{4}‐\text{ratio }$ = 8.450). In contrast, the most significant Z(D) value representing introgression events with IBI breeds was obtained from the trio of (((HFD,BMA),GYR),OUT); D = 0.015; Z(D) = 9.433). In parallel, the Gyr population possessed the highest ${\text{f}}_{4}‐\text{ratio }$ value ((((HFD,BMA),GYR),OUT); ${\text{f}}_{4}‐\text{ratio }$ = 0.154) among those showing IBI introgression. Our results indicated clear evidence of genome-wide level introgression from Hereford and Gyr breeds to the Beefmaster population, which is consistent with the results of PCA and admixture analyses.

**FIGURE 2:**
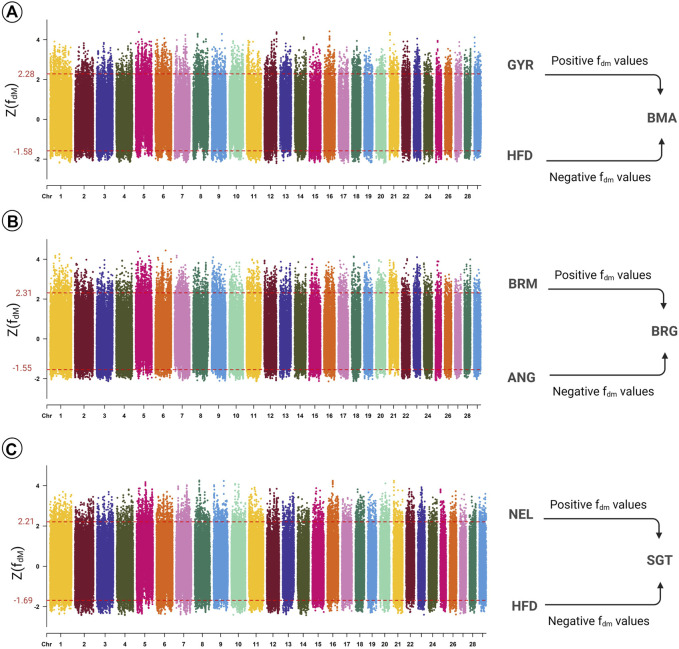
Manhattan plot of the Z-transformed f_dM_ (Z(f_dM_)) at the significance threshold of 5% in Beefmaster **(A)**, Brangus **(B)**, and Santa Gertrudis **(C)**. The f_dM_ > 0 represents regions with Indian *Bos taurus indicus* (IBI) ancestry, while f_dM_ < 0 represents regions with European *Bos taurus* (EBT) origin. Abbreviations of all breeds are given in Table 1.

The Z(f_dM_) values were estimated using the trio of (((HFD,GYR),BMA),OUT). The top 5% genomic regions introgressed from Hereford (*n* = 6,318) and Gyr (*n* = 5,288) into Beefmaster were identified, among which the highest and lowest Z(f_dM_) values were obtained by genomic windows on BTA16 (16:40.21–40.26 Mb; Z(f_dM_) = 4.415) and BTA24 (24:0.10–0.16 Mb; Z(f_dM_) = −2.218), respectively (Figure 2). Regions with f_dM_ > 0 and f_dM_ < 0 comprised 11.7% and 88.3% of the Beefmaster genome. The gene annotation analyses detected 14 and eight candidate genes potentially transferred from Hereford and Gyr ancestry into Beefmaster by the top 5% introgressed genomic regions. The gene enrichment analyses detected three biological pathway terms in which the candidate genes potentially introgressed from Gyr into Beefmaster were significantly over-represented (Supplementary Table S1). In contrast, no significant pathway was detected for candidate genes with Hereford ancestry.

### Brangus

Among the significant Z(D) values, the highest value was obtained by Angus when Charolais was considered as P1 (((CHL,ANG),BRG),OUT); D = 0.087; Z(D) = 29.092). The highest ${\text{f}}_{4}‐\text{ratio }$ among EBT breeds was obtained by the same trio ((((CHL,ANG),BRG),OUT); ${\text{f}}_{4}‐\text{ratio }$ = 2.839). In contrast, the highest Z-score among Z(D) values representing introgression events from IBI to CB breeds was achieved by Brahman ((((HFD,BRG),BRM),OUT); D = 0.015; Z(D) = 6.417). Four trios with ${\text{f}}_{4}‐\text{ratio}$ > 0.1% showed introgression with IBI breeds, out of which the highest value ( ${\text{f}}_{4}‐\text{ratio}=0.220$ ) belonged to Brahman. Our results of Patterson’s D and ${\text{f}}_{4}‐\text{ratio}$ was in parallel with the results of PCA and admixture analyses.

The top 5% genomic regions introgressed from Angus (*n* = 1,198) and Brahman (*n* = 982) into Brangus were identified (Figure 2). The highest and lowest Z(f_dM_) values were obtained by genomic windows on BTA6 (6:77.64–77.66 Mb; Z(f_dM_) = 4.444) and BTA16 (16:34.27–34.28 Mb; Z(f_dM_) = −2.135). Estimation of the f_dM_ of Brangus cattle using the tree topology (((ANG,BRM),BRG),OUT) revealed that the introgressed regions with f_dM_ > 0 and f_dM_ < 0 formed 11.8% and 88.2% of the Brangus genome, respectively. The gene annotation analyses revealed that 581 and 10 candidate genes were potentially transferred by the top 5% introgressed genomic regions from Angus and Brahman into Brangus. Based on the gene enrichment analysis, we found 13 and two biological processes and pathway terms in which the candidate genes introgressed from Angus and Brahman were overrepresented (Supplementary Table S1).

### Santa Gertrudis

Among the significant Z(D) values, the highest value belonged to the Hereford breed (((BRG,HFD), GT),OUT); D = 0.104; Z(D) = 26.542). This result was further confirmed by f_4_-ratio test (((CHL,HFD),SGT),OUT); ${\text{f}}_{4}‐\text{ratio}$ = 10.280). On the contrary, 10 significant Z(D) values were estimated for IBI breeds, among which the Nellore breed obtained the highest Z-score ((((HFD,SGT),NEL),OUT); D = 0.016; Z(D) = 9.830). In agreement with this result, Nellore had the highest value of ${\text{f}}_{4}‐\text{ratio}$ among those representing IBI introgression ( ${\text{f}}_{4}‐\text{ratio}$ = 0.146). Therefore, in agreement to the admixture results, the introgression assessment of Patterson’s D and ${\text{f}}_{4}‐\text{ratio}\;$ clearly depicted the higher levels of genome-wide introgression from Hereford and Nellore into the Santa Gertrudis breed.

The top 5% genomic regions introgressed from Hereford (*n* = 1,237) and Nellore (*n* = 1,085) were determined (Figure 2). The highest and lowest Z(f_dM_) values were obtained by genomic windows on BTA8 (8:8:33.30–33.32 Mb; Z(f_dM_) = 4.264) and BTA14 (14:14:64.37–64.42 Mb; Z(f_dM_) = −2.421). The estimation of the f_dM_ values using the trio of (((HFD,NEL),SGT),OUT) showed that the introgressed regions with f_dM_ > 0 and f_dM_ < 0 contributed to 15.3% and 84.7% of the Santa Gertrudis genome, respectively. The gene annotation analyses revealed that 42 and 15 candidate genes were potentially transferred by the top 5% introgressed genomic regions from Hereford and Nellore into Santa Gertrudis. In total, we identified 12 terms in which the introgressed genes from Nellore to Santa Gertrudis were significantly overrepresented (Supplementary Table S1). One term was also found significant overrepresented by introgressed genes from Hereford to Santa Gertrudis (Supplementary Table S1).

### Recent Signatures of Selection Signals

After obtaining DCMS statistics for 492,954 SNPs, *p*-values were fitted to a normal distribution and corrected for multiple testing. Fourteen, 73, and 85 genomic regions with significant selection signals were found for Beefmaster, Brangus, and Santa Gertrudis, respectively (q-value < 0.05). The average ± standard deviation length of the regions under selection were 24.5 ± 14.1, 17.0 ± 14.9, and 28.5 ± 15.4 Kb in the Beefmaster, Brangus, and Santa Gertrudis breeds, respectively. The total length of regions under significant selection pressure was 299.3, 783.7, and 823.1 Kb in Beefmaster, Brangus, and Santa Gertrudis, respectively. Overall, nine, 24, and 28 candidate genes were identified in the genomic regions underlying selection in the Beefmaster, Brangus, and Santa Gertrudis breeds (Table 2). None of the candidate genes were detected in more than one breed.

**TABLE 2:** Genomic regions detected by the DCMS analyses as being under putative selection in Beefmaster (BMA), Brangus (BRG), and Santa Gertrudis (SGT) breeds.

Chr^a^	Position (Mb)	Q-value	Breed	Gene Id	Associated trait(s) in cattle
BTA2	90.41–90.49	0.001	BRG	*STRADB*	Immune response to intestinal parasite
				*C2CD6*	Marbling score
BTA3	80.75–80.92	0.001	SGT	*JAK1*	Residual feed intake, immune system response to *Mycobacterium bovis*
BTA4	25.26–25.26	0.029	BRG	*AGR2*	Immune response to intestinal parasites, heat tolerance
BTA4	103.31–103.32	0.018	BRG	*KIAA1549*	Conformation
BTA5	36.04–36.05	0.023	SGT	*NELL2*	Puberty
BTA6	81.55–81.56	0.000	BRG	*TECRL*	Puberty, feed conversion ratio
BTA6	103.80–103.81	0.011	SGT	*AFF1*	Puberty
BTA6	104.09–104.13	0.014	SGT	*NUDT9*	Uniformity of yearling weight
BTA7	35.69–35.70	0.017	SGT	*HSD17B4*	Backfat thickness
BTA7	35.83–35.85	0.001	SGT	*TNFAIP9*	—
BTA7	36.01–36.05	0.001	SGT	*DMXL1*	—
BTA7	63.98–64.02	0.030	SGT	*SYNPO*	Meat tenderness
BTA7	64.41–64.44	0.014	SGT	*CCDC69*	—
BTA7	64.72–64.73	0.043	SGT	*FAT2*	Placentation
BTA7	65.07–65.09	0.008	SGT	*GLRA1*	Myoclonus
BTA8	8.96–8.97	0.028	BMA	*MSRA*	Antioxidant defense and lifespan
BTA10	69.77–69.96	0.042	BRG	*EXOC5*	Infectious hoof lesions
				*AP5M1*	Infectious hoof lesions
BTA10	70.85–71.06	0.002	BRG	*ARID4A*	Bull fertility
				*TIMM9*	—
				*KIAA0586*	—
BTA11	10.56–10.58	0.000	BRG	*TET3*	—
BTA11	11.66–11.75	0.000	BRG	*EXOC6B*	Ketosis susceptibility
BTA11	12.92–12.94	0.000	BRG	*DYSF*	Development of the hind quarter, fertility
BTA11	13.55–13.59	0.002	BRG	*CD207*	Ketosis susceptibility
				*CLEC4F*	Ketosis susceptibility
BTA11	14.35–14.38	0.024	BRG	*SRD5A2*	Ketosis susceptibility, sperm motility
BTA11	26.48–26.73	0.038	SGT	*SLC3A*	—
				*PREPL*	Marbling score
				*CAMKMT*	—
BTA11	31.25–31.26	0.049	SGT	*FSHR*	Multiple birth
BTA11	32.16–32.17	0.046	SGT	*NRXN1*	Temperament
BTA11	36.80–36.81	0.008	SGT	*ACYP2*	Carcass and bone weight, feed efficiency, arthrogryposis, macroglossia
BTA11	38.70–38.71	0.011	SGT	*CCDC85A*	—
BTA11	101.28–101.29	0.000	BRG	*LAMC3*	Tick resistance, backfat thickness, conception rate
BTA13	25.46–25.47	0.029	SGT	*KIAA1217*	Gestation length
BTA13	25.80–25.82	0.030	SGT	*ARHGAP21*	Calving-to-first service interval, non-return after 56 days, puberty, post-partum anoestrus
BTA13	26.88–26.96	0.041	SGT	*MY O 3A*	Fertility
				*GAD2*	Fertility, average daily feed intake, meet percent
BTA13	43.59–43.61	0.048	SGT	*UCN3*	Heat tolerance and oxidative stress
BTA13	44.93–44.95	0.003	SGT	*KLF6*	Body conformation, intramuscular fat percentage
BTA13	72.79–72.81	0.030	SGT	*SRSF8*	—
BTA15	62.05–62.06	0.027	BRG	*MPPED2*	Muscle growth and development
BTA15	64.11–64.12	0.003	BRG	*EIF3M*	Fertility
				*CCDC73*	—
BTA15	65.01–65.04	0.002	BRG	*KIAA1549L*	—
BTA15	65.56–65.58	0.027	BRG	*ABTB2*	Slaughter weight
BTA15	67.69–67.70	0.042	BRG	*PRR5L*	Bovine respiratory disease susceptibility, conceptus development
BTA17	10.22–10.23	0.028	BMA	*ARHGAP10*	Intramuscular fat formation
BTA17	29.93–30.01	0.028	BMA	*LARP1B*	—
BTA17	30.09–30.18	0.028	BMA	*ABHD18*	—
				*MFSD8*	-—
BTA17	30.18–30.21	0.028	BMA	*PLK4*	—
BTA17	30.25–30.33	0.043	BMA	*HSPA4L*	Thermal stress
				*SLC25A31*	Semen quality
				*INTU*	—
BTA23	7.29–7.31	0.041	SGT	*COL11A2*	Marbling score, heifer pregnancy
BTA24	21.20–21.21	0.021	SGT	*MOCOS*	Yearling weight
BTA26	47.22–47.23	0.033	BRG	*DOCK1*	Flight speed
BTA29	36.70–36.71	0.029	SGT	*PRDM10*	Post-partum anoestrus

a *Bos taurus* chromosome (BTA).

**FIGURE 3:**
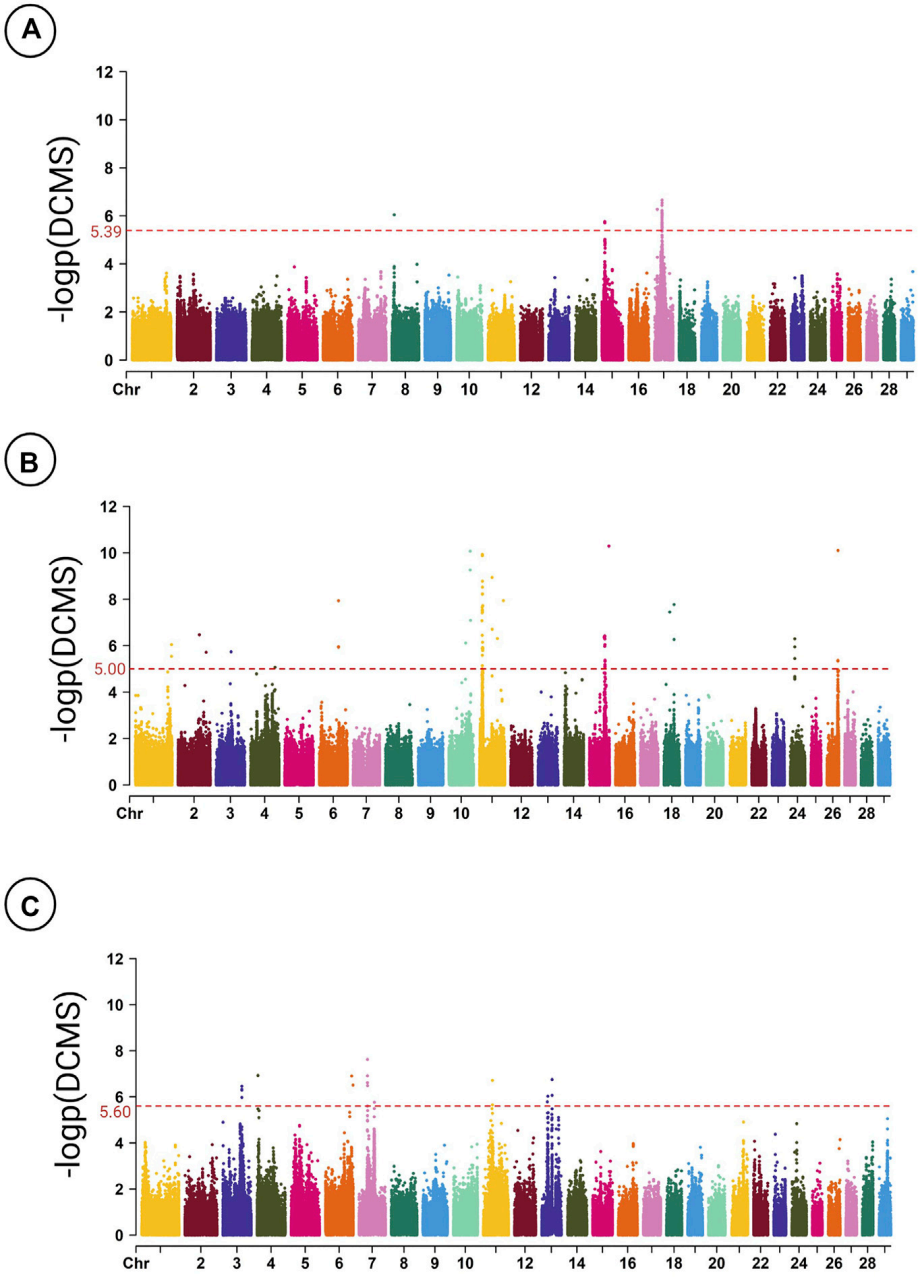
Manhattan plot of the genomic regions under putative selection detected by DCMS in Beefmaster **(A)**, Brangus **(B)**, and Santa Gertrudis **(C)**. The dashed lines represent the significant threshold level at the FDR adjusted *p*-value < 5%.

The distribution of regions under selection across the genome of CB breeds is depicted in Figure 3. The most significant genomic region in Brangus was detected on BTA11 (11:12.91–12.93 Mb; q-value = 0.00002), whereas in Beefmaster, one region on BTA8 and four regions on BTA17 were found with the lowest q-value of 0.028. In Santa Gertrudis, the most significant region is located on BTA7 (7:35.82–35.84 Mb) with q-value = 0.00055.

Gene annotation analysis of the regions underlying selection revealed various previously-reported and novel candidate genes that are associated with a diverse range of traits, including reproduction, susceptibility to infectious diseases, meat quality, thermotolerance, susceptibility to metabolic diseases, genetic diseases, puberty, feed efficiency, carcass quality, sex determination, and temperament (Table 2). In the PPI network analysis, nine, 24, and 26 identifiers were uploaded from the STRING database for Beefmaster, Brangus, and Santa Gertrudis. PPI networks including 28, 33, and 40 genes with significant interactions were constructed for Beefmaster, Brangus, and Santa Gertrudis (Supplementary Figure S1). Gene enrichment analysis revealed that in the Beefmaster breed, the discovered candidate genes were significantly overrepresented in chaperon-mediated protein folding term (GO:0061077), along with three terms related to heat stress response (BTA-2262752, BTA-3371453, bta04141). Regarding the Brangus breed, we found multiple significant pathways associated with immune system function (bta04666, bta04664, bta04662, bta04650), metabolism (BTA-194840, bta04024, GO:0007265), intracellular signaling (bta04014, bta04310, bta04015), and muscle development (bta04810). In the Santa Gertrudis breed, we detected significant metabolic pathways, including lysin (bta00310), beta-alanine (bta00410), pyruvate (bta00620), and arginine and proline (bta00330).

**FIGURE 4:**
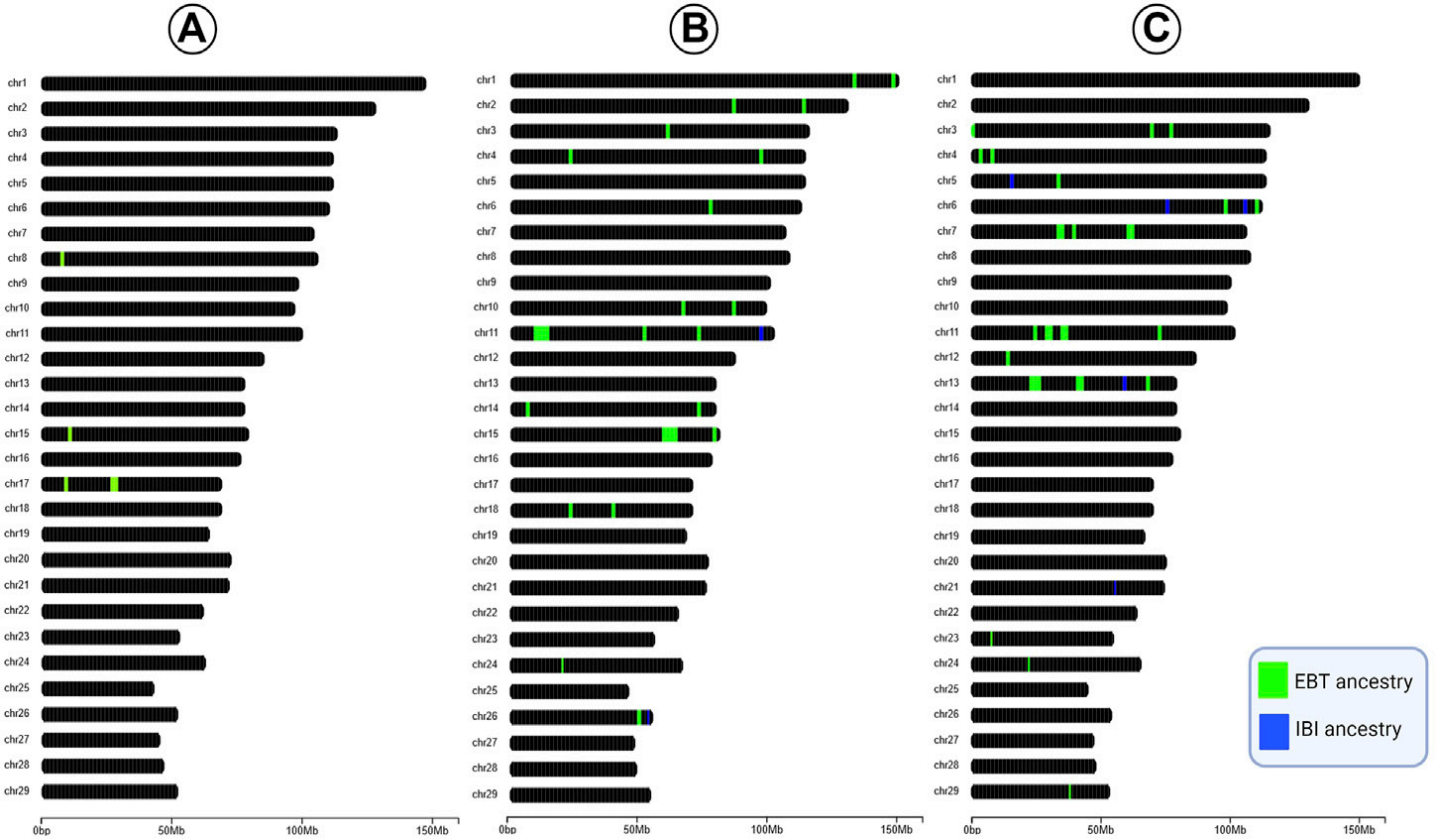
Graphical visualization of the cattle chromosomes with regions underlying significant selection and their origin in Beefmaster **(A)**, Brangus **(B)**, and Santa Gertrudis **(C)**.

Figure 4 shows the overlaps between the results of signatures of selection and introgression analyses. In the Beefmaster breed, all 112 SNPs with significant selection signal had f_dM_ < 0, whereas in Brangus, among 113 significant SNPs identified in DCMS, 104 (92.04%) and nine (7.96%) had f_dM_ < 0 and f_dM_ > 0, respectively. Considering Santa Gertrudis, we found 111 significant markers in DCMS of which 91 (81.98%) and 20 (18.02%) had f_dM_ < 0 and f_dM_ > 0, respectively.

## Discussion

We investigated patterns of ancestry in three *Bos taurus indicus*-influenced CB cattle breeds using high-density genome-wide polymorphism data. Our results confirmed the hybrid nature of three CB cattle breeds of Beefmaster, Brangus, and Santa Gertrudis. We identified signals of indicine and taurine ancestry at genes that are primarily involved in the domestication and adaptation process. Our analyses of the recent signature of selection indicated that genome regions underlying selection pressure among CB breeds are different and are mainly of EBT origin.

### Introgression Events

In neighbor-joining tree and principal component analyses, CB breeds were placed between IBI and EBT populations. The admixture results in K = 2 indicated more indicine genetics (36%) for SGT than BMA (28%) and BRG (30%). In parallel, neighbor-joining tree showed that SGT is closer to indicus ancestry compared with BMA and BRG. Both admixture and phylogenetic analyses were consistent with introgression analysis, which indicated less taurine origin (84.7%) for SGT than that for Brangus (88.2%) and Beefmaster (88.3%). The analyses of admixture, ${\text{f}}_{4}‐\text{ratio},$ and D-statistic clearly showed gene flow from different IBI and EBT breeds to CB populations. Our results were consistent with historical pedigree information of the introgression history of the hybrid cattle breeds (Ritchie, 2009). Using D, ${\text{f}}_{4}‐\text{ratio}$ , and f_dM_ statistics, we observed that, on average, only 12.9% of the studied CB breeds’ genome was introgressed from indicine origin, which is in agreement with the results reported by McTavish et al. (2013), who indicated that only 11% of recently formed hybrid breeds’ genome is of indicine ancestry. Moreover, Barbato et al. (2020) reported that approximately 11–13% of indicine ancestry were recorded in three Central Italian breeds of Marchigiana, Romagnola, and Chianina. Our admixture results showed a higher level of IBI introgression (average of 32.0%) in the studied CB population. The explanation for this discordance might be that a historical model is not fitted in admixture analysis, and it is unrealistically assumed that all populations have derived from a single ancestral group (Alexander et al., 2009). On the contrary, D, ${\text{f}}_{4}‐\text{ratio}$ , and f_dM_ statistics include fitting a simple phylogenetic tree model to a group of populations, and they provide a formal test for a history of admixture in that context (Patterson et al., 2012). Therefore, a more accurate estimation of introgression would be feasible (Malinsky et al., 2021).

Using the f_dM_ statistics we inferred genomic local ancestry in CB breeds. In all chromosomes, some segments ranked the highest 95 percentile or the lowest 95 percentile. Barbato et al. (2020) identified multiple introgressed genomic regions in three Italian breeds in all chromosomes except in BTA17 and BTA 28. However, they only searched for indicine-driven introgressed regions. Five genes were common among the candidate genes with EBT ancestry (Supplementary Figure S2). Among them, Neurexin 3 (*NRXN3*) is involved in the genetic architecture of cattle temperament (Paredes-Sánchez et al., 2020). *NRXN3* is a member of the Neurexins family that are cell adhesion molecules in the nervous system to specify and stabilize excitatory and inhibitory synapses (Aoto et al., 2013). Another gene from this family, Neurexin 1 (*NRXN1*), was shared between candidate genes with EBT ancestry in Brangus and Santa Gertrudis. This gene has been proposed as a candidate gene for a moderate temperament of cattle (Qanbari et al., 2014). The *Bos taurus indicus* crosses, particularly Brahman crosses, have been reported to be more temperamental than EBT breeds but less aggressive/fearful than IBI breeds (Hoppe et al., 2010). Therefore, the inheritance of alleles with EBT ancestry may contribute to less fearful or excitable animal’s response to handling or forced movement by humans. Another candidate gene, TSC22 Domain Family Member 1 (*TSC22D1*), encodes a member of the TSC22 domain family of leucine zipper transcription factors and belongs to the large family of early response genes, which are activated rapidly in response to a wide range of cellular stimuli (Kester et al., 1999). This gene has been found to be associated with maternal lipomatous myopathy (Peletto et al., 2017) and calving difficulty (Purfield et al., 2020) in cattle. It has been generally assumed that IBI cattle have less calving difficulty than EBT breeds due to their pelvic structure. However, the greater emphasis on growth rate has increased the risk of calving difficulties in CB breeds (Morrison et al., 1989). Two candidate genes of CASP2 and RIPK1 Domain Containing Adaptor with Death Domain (*CRADD*) and Sodium/Potassium Transporting ATPase Interacting 2 (*NAKIN2*) were shared among candidate genes with IBI ancestry. *CRADD* encodes a death domain-containing protein that can induce cell apoptosis (Ahmad et al., 1997). This gene is involved in cattle embryonic development, a process in which apoptosis plays a critical role in adapting myometrial cells during pregnancy (Rehman et al., 2003). In contrast, the function of *NAKIN2* in cattle is not comprehensively understood yet.

In all CB populations, gene enrichment study of genes with IBI ancestry showed the detected candidate genes were significantly involved in biological processes associated with apoptosis regulation by tumor suppressor TP53 (Supplementary Table S1). TP53 is upregulated in response to several stimuli, including activation of oncogenes, DNA damage, or nutrient deprivation (Aubrey et al., 2018). Subsequently, apoptosis will be induced through intrinsic or extrinsic pathways (Aubrey et al., 2018). The *TP53* gene and its pathways are associated with some traits in cattle, such as heifer fertility (Neupane et al., 2017) and carcass weight (Carvalho et al., 2019). Carcass weight and quality are usually higher in EBT breeds than IBI’s (Highfill et al., 2012). Indeed, cattle with IBI influence tend to have lower meat tenderness and growth rate than EBT (Wheeler et al., 2010; Elzo et al., 2012). Therefore, increases in the proportion of alleles with EBT origin in the crossbred progeny could result in higher meat quality and yield. Santa Gertrudis and Beefmaster genes with IBI ancestry are significantly involved in the Class C/3 (Metabotropic glutamate/pheromone receptors) pathway. Cattle pheromones are crucial in reproduction and social behaviors, e.g., sexual attraction, mother-young interactions, estrus indication, puberty acceleration, reducing the post-partum anestrus, hormonal stimulation, and erection (Mucignat-Caretta, 2014). Therefore, they could be critical factors in animals’ fitness and reproductive success.

Santa Gertrudis candidate genes with EBT ancestry were significantly overrepresented in the neural crest differentiation process (Supplementary Table S1). Neural crest cells have the potential to differentiate into several different cell types of the vertebrate body. They are the origin of most parts of the peripheral nervous system, endocrine cells in the adrenal medulla and thyroid, the frontal part of the head, and parts of the cardiovascular system (for a review, please see Dupin et al., 2006). It has been hypothesized that a reduction in neural crest cell proliferation and migration is an essential genetic mechanism of early domestication (Wilkins et al., 2014). Moreover, further changes to the neural crest may potentiate the later evolution of other domestication traits (Wright et al., 2020). Oxidation by cytochrome P450 was the most significant pathway in which Brangus candidate genes with EBT ancestry were overrepresented. Overall, five genes were overrepresented in the Oxidation by cytochrome P450 WikiPathway term which includes 39 genes in cow. Drugs, toxins, and many foreign hydrophobic compounds are removed from the body by cytochrome P450 enzyme system, particularly in the liver (Moubarak & Rosenkrans, 2000). Moreover, the critical role of P450 genes in oxidative stress, inflammatory-based diseases, and synthesis and metabolism of sterols, steroid hormones, and lipid biofactors such as eicosanoids, vitamin D3, and retinoids in mammals is well-documented (Omura, 1999; Kuhn et al., 2020). Therefore, understanding the inheritance of cytochrome P450 oxidation functional elements from EBT breeds might be essential in veterinary practice, nutrition, and metabolic priorities of CB breeds.

### Recent Signatures of Selection

To the best of our knowledge, this study is the first report of signatures of selection in the Beefmaster breed. In this breed, DCMS found three locations on the bovine genome with the most significant peaks (q-value = 0.02809), including two locations on BTA17 (17:10.22–10.23 Mb and 17:29.93–30.18 Mb) and one on BTA8 (8:8.97–8.97 Mb) (Figure 3). We found six candidate genes in these regions, including Methionine Sulfoxide Reductase A (*MSRA*), Rho GTPase Activating Protein 10 (*ARHGAP10*), Polo Like Kinase 4 (*PLK4*), La-related protein 1 (*LARP1B*), Abhydrolase Domain Containing 18 (*ABHD18*), and Major Facilitator Superfamily Domain Containing 8 (*MFSD8*), of which the function of the last three genes in CB breeds are not comprehensively known. Oxidation of proteins by reactive oxygen species can occur in oxidative stress, such as heat stress and several diseases. Free and protein-bound methionine residues are susceptible to oxidation to methionine sulfoxide derivatives (Moskovitz et al., 2001). However, *MSRA* can repair this modification by catalyzing the thioredoxin-dependent reduction of free and protein-bound Met(O) to methionine (Moskovitz et al., 2001). Therefore, *MSRA* plays a vital role in stress response in mammals. *ARHGAP10* is also a member of the Rho-GTPase activating protein (Rho-GAP) family, regulating Rho-GTPase signaling pathways, and these pathways are involved in actin cytoskeleton dynamics, cell proliferation, and differentiation (Bassères et al., 2002). A recent study on Japanese black cattle indicated that this gene might be involved in intramuscular fat formation (Ueda et al., 2021). The PPI network analysis of Beefmaster candidate genes indicated the critical role of Heat Shock Protein Family A Member 4 Like (*HSPA4L*) with contribution into three biological pathways (Supplementary Figure S1). *HSPA4L* is a member of the HSP70 gene family, the largest and the most conserved protein family throughout evolution (Daugaard et al., 2007). HSP70 genes function in heat tolerance, protection of cells against apoptosis, and reactive oxygen species (Feder & Hofmann, 1999). A recent transcriptome study in cattle revealed that the expression of *HSPA4L* is upregulated in the milk somatic cells during heat challenge (Garner et al., 2020). Gene enrichment analysis revealed that Beefmaster candidate genes were significantly overrepresented in pathways related to post-translation protein modifications and stress response (Supplementary Figure S1). It is noteworthy that Beefmaster has been widely used not only for their heat tolerance and adaptability but excellent growth and carcass quality (Cartwright, 1970; Wheeler et al., 2010).

Regarding the Brangus breed, we identified 73 genomic windows underlying selection, of which the most significant signal was found on BTA11 (11:12.92–12.94 Mb; q-value = 1.02E-06) (Figure 3). The well-known candidate gene of Dysferlin (*DYSF*) is located in this region. Dysferlin, known as dystrophy-associated fer-1-like protein, is encoded by the *DYSF* gene (Blandin et al., 2012). This calcium-dependent transmembrane protein is mainly expressed in skeletal and cardiac muscles to enhance calcium-mediated membrane fusion and sarcolemmal repair (Blandin et al., 2012). Mutations in this gene cause multiple different phenotypes of muscular dystrophies in humans (Vilchez et al., 2005). In contrast, studies in cattle demonstrated its strong association with the development of cattle muscularity (Doyle et al., 2020), sire conception rate (Rezende et al., 2018), and female reproductive performance (Fonseca et al., 2020). A recent study conducted by Zhang et al. (2020) indicated that this gene might be under strong positive selection for high altitudes adaptation in Chinese indigenous cattle. We could not find any overlapping genomic regions between our results and a recently-conducted signatures of selection study in the Brangus breed by Paim et al. (2020). The reason could be that we investigated recent signatures of selection, i.e., those regions underlying selection following the breed formation, using multiple EHH-based methods. While runs of homozygosity, which is an indicator of genomic autozygosity, may arise due to several population phenomena like inbreeding, genetic drift, consanguineous mating, population bottleneck, as well as natural and artificial selection (Falconer and Mackay, 1996; Curik et al., 2014).

PPI network analysis of Brangus candidate genes revealed significant interactions with the Rac subfamily of the Rho family of GTPases, particularly RAC1, RAC2, and RAC3. Moreover, in PPI network analysis, we found multiple pathways which are connected to these genes, including Ras signaling pathway, regulation of actin cytoskeleton, Rho GTPase cycle, Rap1 signaling pathway, cAMP signaling pathway, and Ras protein signal transduction. Ras signaling is a critical intracellular signaling pathway that plays a vital role in cellular proliferation and differentiation, survival, and gene expression (Vojtek and Der, 1998). Rho GTPases of the Ras superfamily and Rap1 protein act as molecular switches to control a wide range of essential biochemical pathways in response to environmental stimuli through intracellular signal transduction pathways (Bar-Sagi and Hall, 2000). It is noteworthy that Rap1 activation is mediated by several second messengers, such as cAMP (Zwartkruis and Bos, 1999). Studies on cattle have shown the importance of Ras signaling pathway in meat quality (Li et al., 2020) and subcutaneous fat deposition (Taniguchi et al., 2008), Rap1 signaling pathway in muscle development (Zhan et al., 2018), and cAMP signaling pathway in lipid metabolism (Junjvlieke et al., 2020).

To the best of our knowledge, signatures of selection analysis have not been previously conducted on Santa Gertrudis. We identified 85 genomic regions with significant selection signals in this breed (Figure 3). The most significant region was located on BTA7 (7:35.83–35.85 Mb; q-value = 0.00055). We found a candidate gene of Tumor Necrosis Factor-Alpha Inducible Protein 9 (*TNFAIP9*) in this region. *TNFAIP9* is a member of the STEAP (six transmembrane epithelial antigen of prostate) family vital for cellular iron uptake and homeostasis (Han et al., 2018). Studies in humans suggested that *TNFAIP9* might be involved in adipocyte development and metabolism (Gui et al., 2016). However, its function in cattle has not been comprehensively understood yet. We also detected several genomic regions with significant selection signals on BTA11 (11:31.25–38.71 Mb) and BTA13 (13:25.80–44.95 Mb). In gene annotation study of these regions, several candidate genes have been found related to reproductive system function in cattle, including Follicle Stimulating Hormone Receptor (*FSHR*) (Widmer et al., 2021), *KIAA1217* (Mohammadi et al., 2020), Rho GTPase Activating Protein 21 (*ARHGAP21*) (Wolf et al., 2021), Myosin IIIA (*MY O 3A*) (Mohammadi et al., 2020), and Glutamate Decarboxylase 2 (*GAD2*) (Mohammadi et al., 2020). Fertility is an essential element in beef cattle production because it directly relates to producing the offspring necessary to offset costs in production systems (Thundathil et al., 2016). This trait has been improved in CB breeds during the last decades through assisted reproductive technologies of artificial insemination and genetic selection (Cammack et al., 2009). We also detected a significant signal on BTA 3 (3: 80.75–80.92 Mb). A larger overlapped region has been previously detected to be under significant selection in the Sheko breed (3:80.10–80.93 Mb) (Bahbahani et al., 2018). We detected the candidate gene of Janus Kinase 1 (*JAK1*) in this region which is involved in cattle residual feed intake (Xi et al., 2015) and immune response to *Mycobacterium bovis* (Imai et al., 2003).

The PPI network analysis revealed the central role of two members of the aldehyde dehydrogenase (ALDH) gene superfamily, including Aldehyde Dehydrogenase 3 Family Member A2 (*ALDH3A2*) and Aldehyde Dehydrogenase 7 Family Member A1 (*ALDH7A1*). Recent studies have indicated the role of *ALDH7A1* in feed efficiency (De Oliveira et al., 2014) and growth rate (Buzanskas et al., 2014), but less information is available about the *ALDH3A2* role. Gene enrichment analysis indicated that Beefmaster candidate genes were significantly overrepresented in several metabolic pathways, particularly of amino acids metabolism (Supplementary Figure S1). Amino acids are the principal nutrition component for protein synthesis and meat, and rapid rates of muscle protein deposition is positively correlated with growth and efficient beef production. Lysin is a limiting amino acid for optimizing the growth of certain animals such as pigs and poultry, and it is one of the first three limiting amino acids (methionine, lysine, and threonine) in growing cattle diet (Richardson and Hatfield, 1978). Moreover, lean growth rate in cattle is positively associated with overall feed efficiency in growing and finishing animals (Archer et al., 1997). Therefore, the lysin metabolism might play an important role in selection of feed efficient animals with high growth rate.

### Integration of Recent Signatures of Selection and Introgression Events

The overlap between the results of signatures of selection and introgression analyses deciphered that the selected regions in studied CB breeds were predominantly EBT in origin (Figure 4). However, the proportion of EBT ancestry in selected regions is higher in Beefmaster (100%) than Brangus (92.04%) and Santa Gertrudis (81.98%). Our results complement the previous study of Paim et al. (2020) that showed that homozygous regions in Brangus are mainly of Angus ancestry. Our results show how the combination of selection and complementary can shift the genetic architecture of CB populations following the breed formation.

Although a reasonable number of genotyped animals were available for this study, the sample size is still a limiting factor. Therefore, larger datasets could enable accurate genotype imputation analyses and therefore, the inclusion of a larger number of SNP markers in the analyses. In addition, it would be recommended to add other breeds that might have contributed to the formation of the three CB analyzed as well as other composite populations such as the Montana tropical Composite (Grigoletto et al., 2020).

## Conclusion

Our study revealed human-mediated introgression events and genomic regions underlying selection in three CB breeds. We confirmed the low contribution of alleles with IBI origin in the CB cattle genome. The majority of selected genomic regions in CB cattle breeds came from EBT that can be in conjunction with the traits of interest for genetic improvement and selection. Our results demonstrate how complementarity and selection collaborate in shaping the genetic architecture of the CB breeds population. We showed that the overlaps between these two events were breed-specific, suggesting that differences in breeding objectives and selection intensities exist between CB breeds. Investigating the CB breeds’ genomic architecture allows the estimation of genome-wide indicine and taurine genome proportions and demonstrates the locations within the genome where alleles with either taurine or indicine origin provide a selective advantage. Such findings provide the opportunity to control the breeding programs more efficiently.

## Data Availability

Publicly available datasets were analyzed in this study. This data can be found here: http://widde.toulouse.inra.fr/widde/widde/main.do?module=cattle.

## Abbreviations

BMA, beefmaster; BRM, brahman; BRG, brangus; CHL, charolais; CB, composite beef cattle; DCMS, de-correlated composite of multiple signals; EBT, european *Bos taurus taurus*; EHH, extended haplotype homozygosity; FDR, false discovery rate; GOBP, gene ontology biological process; GYR, gyr; HFD, hereford; IBI, indian *Bos taurus indicus;* iHH12, integrated haplotype homozygosity pooled; iHS, integrated haplotype score; KEGG, kyoto encyclopedia of genes and genomes; NEL, nellore; OUT, outgroup (yak); PMT, piedmontese; PPI, protein-protein interaction; SGT, santa gertrudis; SNP, single nucleotide polymorphism; Z(D), Z-transformed D; Z(f_dM_), Z-transformed f_dM_.
