# Supplementary figures and images for: Genome-Wide Selection Signatures and Human-Mediated Introgression Events in Bos taurus indicus-influenced Composite Beef Cattle
Source: Front Genet. 2022 May 30;13:844653. doi: 10.3389/fgene.2022.844653 (PMC9201998; doi:10.3389/fgene.2022.844653)

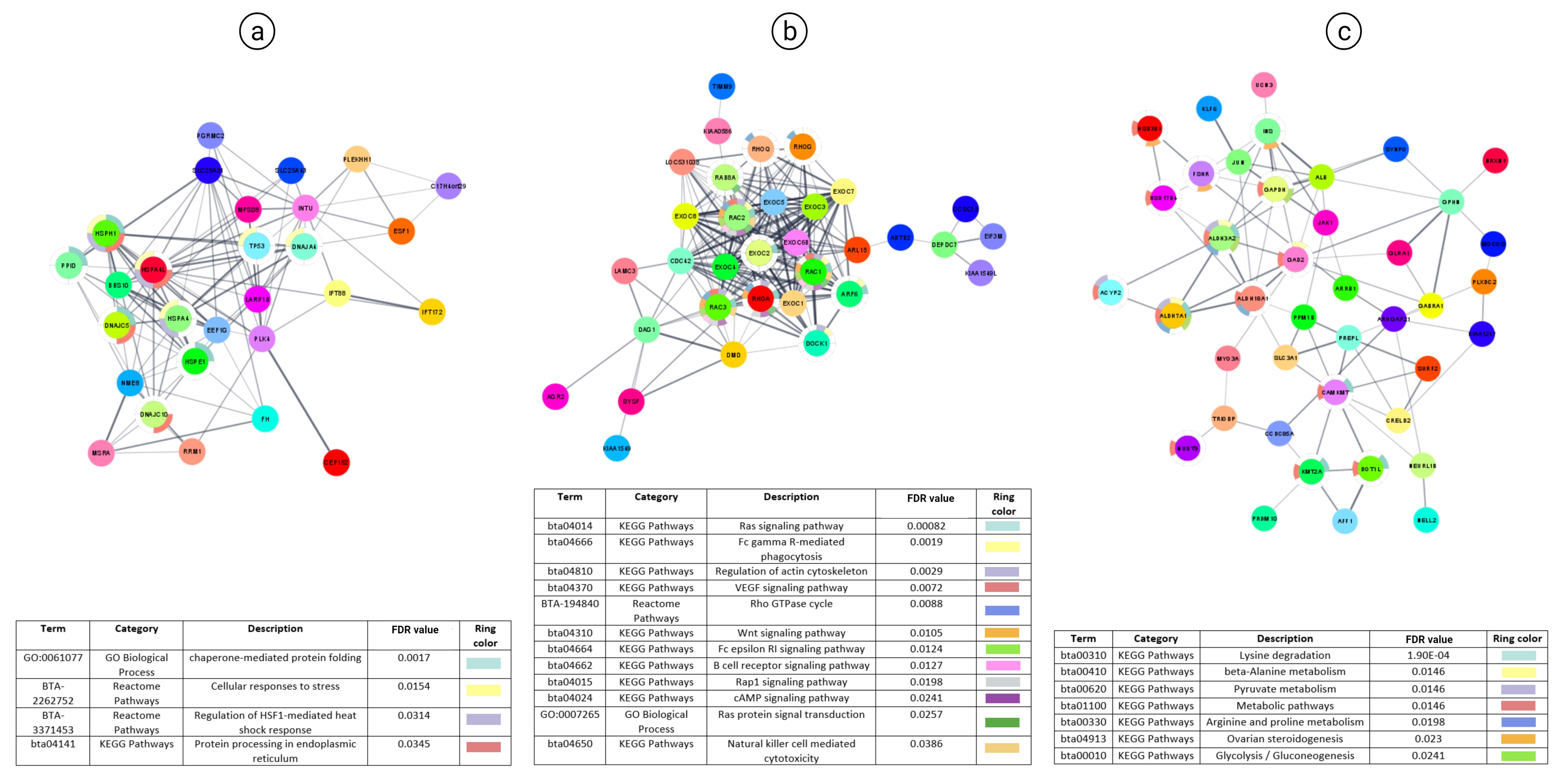

Supplement: Supplementary file 1 [file Image1.JPEG]

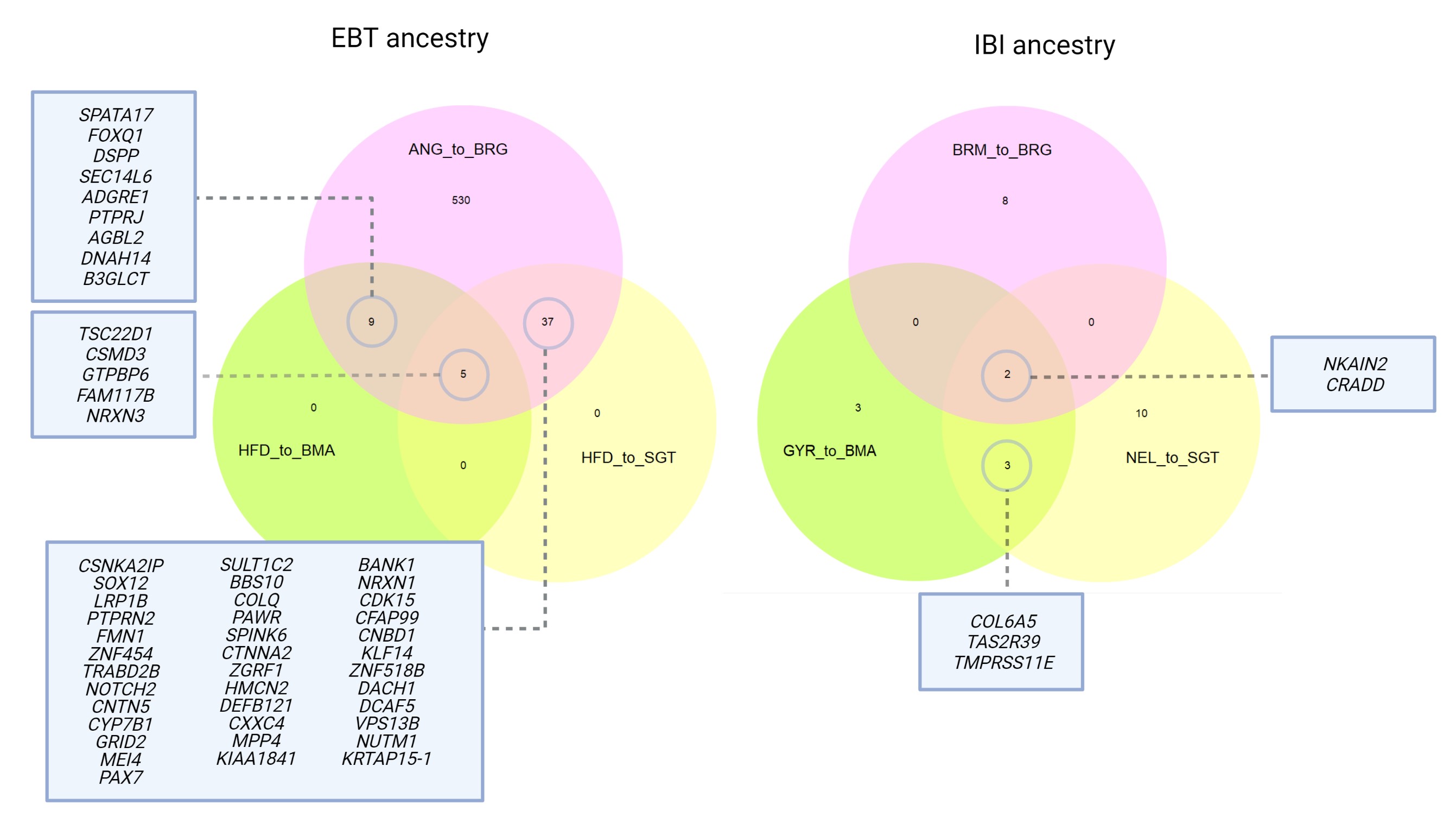

Supplement: Supplementary file 2 [file Image2.JPEG]
