# Supplementary material for: Genome-Wide Selection Signatures and Human-Mediated Introgression Events in Bos taurus indicus-influenced Composite Beef Cattle
Source: Front Genet. 2022 May 30;13:844653. doi: 10.3389/fgene.2022.844653 (PMC9201998; doi:10.3389/fgene.2022.844653)
Supplement: Supplementary file 3 [file DataSheet1.docx]

**Appendices**

**Table S1.** Gene enrichment of candidate genes with European *Bos taurus* (EBT) or Indian *Bos indicus* (IBI) ancestry introgressed to composite beef cattle breeds. Abbreviations of breeds are given in Table 1.

| Term | Category | Description | Term size | Intersection size | FDR^1^ value | From^2^ | To^3^ |
| --- | --- | --- | --- | --- | --- | --- | --- |
| WP:WP1077 | WP^4^ | Oxidation by Cytochrome P450 | 39 | 5 | 0.000 | ANG | BRG |
| REAC:R-BTA-420499 | REAC^5^ | Class C/3 (Metabotropic glutamate/pheromone receptors) | 24 | 2 | 0.005 | NEL | SGT |
| REAC:R-BTA-6803207 | REAC | TP53 Regulates Transcription of Caspase Activators and Caspases | 4 | 1 | 0.005 | BRM | BRG |
| REAC:R-BTA-5633008 | REAC | TP53 Regulates Transcription of Cell Death Genes | 11 | 1 | 0.008 | BRM | BRG |
| WP:WP3273 | WP | Neural Crest Differentiation | 84 | 2 | 0.009 | HFD | SGT |
| WP:WP1006 | WP | Meta-pathway biotransformation | 112 | 5 | 0.013 | ANG | BRG |
| GO:0001580 | GO:BP^6^ | Detection of chemical stimulus involved in sensory perception of bitter taste | 31 | 2 | 0.016 | NEL | SGT |
| GO:0050912 | GO:BP | Detection of chemical stimulus involved in sensory perception of taste | 36 | 2 | 0.016 | NEL | SGT |
| GO:0050913 | GO:BP | Sensory perception of bitter taste | 35 | 2 | 0.016 | NEL | SGT |
| WP:WP3248 | WP | Tryptophan metabolism | 42 | 3 | 0.018 | ANG | BRG |
| WP:WP970 | WP | Fatty Acid Omega Oxidation | 11 | 2 | 0.018 | ANG | BRG |
| WP:WP1053 | WP | Estrogen metabolism | 11 | 2 | 0.018 | ANG | BRG |
| WP:WP3226 | WP | Aryl Hydrocarbon Receptor | 35 | 3 | 0.018 | ANG | BRG |
| WP:WP3163 | WP | Estrogen Receptor Pathway | 13 | 2 | 0.021 | ANG | BRG |
| WP:WP3264 | WP | Tamoxifen metabolism | 14 | 2 | 0.021 | ANG | BRG |
| REAC:R-BTA-6803207 | REAC | TP53 Regulates Transcription of Caspase Activators and Caspases | 4 | 1 | 0.022 | GYR | BMA |
| WP:WP3157 | WP | Pathogenic Escherichia coli infection | 53 | 3 | 0.023 | ANG | BRG |
| GO:0050909 | GO:BP | Sensory perception of taste | 54 | 2 | 0.028 | NEL | SGT |
| REAC:R-BTA-388396 | REAC | GPCR downstream signaling | 342 | 3 | 0.029 | NEL | SGT |
| REAC:R-BTA-6803207 | REAC | TP53 Regulates Transcription of Caspase Activators and Caspases | 4 | 1 | 0.029 | NEL | SGT |
| REAC:R-BTA-5633008 | REAC | TP53 Regulates Transcription of Cell Death Genes | 11 | 1 | 0.030 | GYR | BMA |
| REAC:R-BTA-372790 | REAC | Signaling by GPCR | 414 | 3 | 0.030 | NEL | SGT |
| GO:0006357 | GO:BP | Regulation of transcription by RNA polymerase II | 2,033 | 80 | 0.032 | ANG | BRG |
| GO:0007156 | GO:BP | Homophilic cell adhesion via plasma membrane adhesion molecules | 110 | 12 | 0.032 | ANG | BRG |
| GO:0006366 | GO:BP | Transcription by RNA polymerase II | 2,116 | 81 | 0.036 | ANG | BRG |
| REAC:R-BTA-420499 | REAC | Class C/3 (Metabotropic glutamate/pheromone receptors) | 24 | 1 | 0.043 | GYR | BMA |
| WP:WP3209 | WP | Arylhydrocarbon receptor (AhR) signaling pathway | 23 | 2 | 0.046 | ANG | BRG |
| REAC:R-BTA-1461957 | REAC | Beta defensins | 17 | 1 | 0.046 | NEL | SGT |
| REAC:R-BTA-1461973 | REAC | Defensins | 17 | 1 | 0.046 | NEL | SGT |
| REAC:R-BTA-418594 | REAC | G alpha (i) signaling events | 201 | 2 | 0.046 | NEL | SGT |
| REAC:R-BTA-5633008 | REAC | TP53 Regulates Transcription of Cell Death Genes | 11 | 1 | 0.046 | NEL | SGT |

**^1^**False discovery rate

**^2^**Candidate genes introgressed from this breed.

^3^Candidate genes introgressed to this breed.

**^4^**WikiPathway

**^5^**Reactome pathway

**^6^**Gene ontology biological process

**Figure S1.** PPI network and functional enrichment of candidate genes underlying selection obtained by application of *stringApp* of *Cytoscape* in Beefmaster (a), Brangus (b), and Santa Gertrudis (c).

**Figure S2.** Overlapped candidate genes with European *Bos taurus taurus* (EBT) or Indian *Bos taurus indicus* (IBI) ancestry studied composite beef breeds. Abbreviations of breeds are given in Table 1.
